# Prevalence and types of thyroid malignancies among thyroid enlarged patients in Gondar, Northwest Ethiopia: a three years institution based retrospective study

**DOI:** 10.1186/1471-2407-14-899

**Published:** 2014-12-02

**Authors:** Tadele Melak, Biniam Mathewos, Bamlaku Enawgaw, Debasu Damtie

**Affiliations:** Department of Clinical Chemistry, School of Biomedical and Laboratory Sciences, College of Medicine and Health Sciences, University of Gondar, Gondar, Ethiopia; Department of Immunology and Molecular Biology, School of Biomedical and Laboratory Sciences, College of Medicine and Health Sciences, University of Gondar, Gondar, Ethiopia; Department of Hematology, School of Biomedical and Laboratory Sciences, College of Medicine and Health Sciences, University of Gondar, Gondar, Ethiopia

**Keywords:** Gondar, Prevalence, Thyroid malignancy, Types of thyroid malignancy

## Abstract

**Background:**

Thyroid carcinoma is the leading cause of death among endocrine cancers second to carcinoma of the ovary. Now a day, the incidence of thyroid malignancy is increasing more rapidly than any other malignancy. But data on the prevalence of thyroid malignancy among thyroid enlarged patients were very limited in the study area. Therefore, this study was aimed to determine the prevalence of thyroid malignancies among thyroid enlarged patients.

**Methods:**

Data of 846 thyroid enlarged patients registered from January 2010 to February 2013 were collected from fine needle aspirate cytology and histology (for inconclusive and the neoplastic cases) log books. It was entered and analyzed using SPSS version 20. Odds ratio was calculated to assess the presence and strength of association between the outcome variable and the explanatory variables. P-values less than 0.05 were considered statistically significant.

**Results:**

Among the 846 thyroid enlarged patients, 62(7.3%) were confirmed to have malignancy. Among malignancies papillary thyroid carcinoma was the leading, 28 (45.2%), followed by follicular thyroid carcinoma, 18 (29%), and the least type of thyroid malignancies were medullary thyroid carcinoma and hurtle cell carcinoma, each accounts 1 (1.6%). Severe form of thyroid malignancy, undifferentiated thyroid carcinoma, was also accounted significant proportion, 12.9%. Older patients having an age of greater than 60 years and patients with solitary thyroid enlargement were more affected by malignancy compared to the reference age group, 11–20 years and diffused type of enlargement respectively (AOR: 10.96 (3.15-38.1; AOR: 8.82 (3.49-22.32) respectively).

**Conclusions:**

The prevalence of thyroid malignancy was significantly high and the leading type of malignancy was papillary thyroid carcinoma followed by follicular thyroid carcinoma. Thyroid malignancy was found to have statistically significant association with type of enlargement and age.

## Background

Worldwide, the overall prevalence of thyroid malignancy is approximately 1–5% of all cancers in women and less than 2% in men. During the past several decades, an increasing incidence of thyroid cancer has been reported in European countries [1–3], USA [4] and Canada [5]. It is now the fastest growing cancer type and the sixth most common cancer [1, 3, 6].

In Europe alone, thyroid malignancy affects approximately 24,826 individuals annually, with an estimated mortality rate of 5,993 patients each year [7]. It is also one of the thyroid diseases problems in sub Saharan Africa due to high prevalence of iodine deficiency goiter. For instance, the iodine deficiency is suggested to play a role for follicular cancer increment in South Africa [8]. It is the cause of significant mortality and morbidity of patients, particularly from undifferentiated thyroid carcinoma. Death from differentiated thyroid carcinoma, however, may also occur unless early diagnosis and treatment is initiated [9].

To design and implement cost effective and appropriate intervention, knowledge on local prevalence and distribution of thyroid malignancies among thyroid enlarged patients have paramount importance. In the study area there is scarcity of information on prevalence and type of thyroid malignancies. Hence, this study is aimed to determine the prevalence and types of thyroid malignancies among thyroid enlarged patients in Gondar, Northwest Ethiopia.

## Methods

Institutional based retrospective study was conducted among 846 thyroid enlarged patients registered at Gondar University referral Hospital (GUH) from January 2010 to February 2013. The Hospital is located in Gondar town which is the capital city of North Gondar administrative zone of Amhara regional state, Ethiopia. The Hospital serves a population of around five million across Amhara region and adjacent regions. In the hospital conventional fine needle aspirate cytology (FNAC) technique without any ultrasound guidance has been used for the assessment of thyroid enlargement. Usage of ultrasound is unusual to suggest thyroid malignancy except in case of thyroid cyst.

Demographic information of patients, clinical features, FNAC and tissue biopsy results were extracted from FNAC and tissue biopsies log book of Pathology department using data abstraction sheet. For the current study, FNAC results were categorized under 4 groups. When FNAC results had reported as “papillary thyroid carcinoma (PTC)”, “medullary thyroid carcinoma (MTC)”, “undifferentiated carcinoma”, “lymphoma” or “metastatic tumor”, the author categorized under malignant group. The authors also classified them as neoplasm lesion if the reports had been “neoplasm” or “suggestive for neoplasm”. Likewise, if the reports had been “colloid goiter”, “thyroid cyst” and “thyroiditis”, it was reported as benign (non-neoplastic). Moreover, if it had reported as “non diagnostic” or “suggestive for malignancy”, the author classified them as inconclusive FNAC result. The neoplasm and the inconclusive FNAC results were confirmed by the tissue biopsy findings.

The size and type of enlargements were determined previously by physicians. The size had been assessed based on the measurement of the enlargements by meter in a two dimensional fashion and the diameter of the enlargement was estimated from the area of the enlargement. The type of enlargement was classified as diffuse: if the most part of the gland enlarged in a consistent manner, multi-nodular: if there was more than one nodule by clinical palpation, and solitary if there was a single nodule in either of the thyroid lobules.

Data were entered and analyzed using SPSS version 20. Descriptive statistical analyses were done to give a clear picture of background variables like age, sex and clinical data. Odds ratio was calculated to assess the presence and strength of association between outcome variable and explanatory variables. Ethical approval was obtained from Research and Ethics Committee of School of Biomedical and Laboratory Sciences. Confidentiality was maintained while collecting data using codes.

## Results

Among a total of 846 thyroid enlarged patients 661(78.1%) were females and 185 (21.9 %) were males with male to female ratio of 1:3.6. The mean age at diagnosis was 29.7 year for males (ranges from2-78 years SD; 16.5) and 30.8 year for females (ranges from 5–80 years; SD: 14). Majority of the study participants were at the age of 21–30 years 257/846 (30.4%) followed by 11–20 years of age, 238/846 (28.1%).

The types of thyroid enlargements observed in this study were multi nodular goiter (MNG), diffuse and solitary with a proportion of 59.3%, 29% and 11.7% respectively. The duration of enlargement found to be long in most of the study participants. The mean duration and enlargement diameter at the time of diagnosis were 5.32 years (range from 1 week to 50 years) and 6.1 cm (ranges between1.13 cm -19.54 cm) respectively. Most of study participants (96.9%) had a thyroid enlargement of 3.5 cm diameter and more, in size.

In this study, the prevalence of thyroid malignancy was 7.3% (62/846). Result from FNAC demonstrated thyroid malignancies among 34 cases, follicular and hurtle cell neoplasms among 54 cases, inconclusive for malignancies among12 cases and the remaining 759 were nodular colloid goiters thyroid cysts and thyroiditis. Of the 54 follicular neoplasms diagnosed by FNAC, 19 (35.2%) cases were malignant, while among 12 cases with an inconclusive FNAC report, 9(75%) revealed malignant cancer on the final histopathological examination.

**Figure 1:**
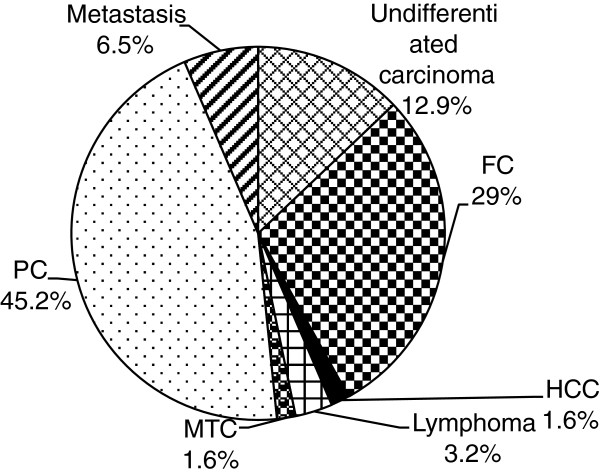
**Type of thyroid malignancies at pathology laboratory of GUH from 2010–2012.** From a total of 62 malignant cases papillary carcinoma (PTC) accounts highest 28/62 (45.2%) followed by follicular carcinoma (FTC) 18/62 (29%). Medullary thyroid carcinoma (MTC) and hurthle cell carcinoma (HCC) accounts one (1.6%).

The leading type of malignancy was PTC 28/62 (45.2%) followed by follicular thyroid carcinoma (FTC) 18/62 (29%). and the least type of thyroid malignancies were MTC and hurthle cell carcinoma (HCC), each accounts 1/62 (1.6%). Hurtle cell carcinoma and MTC were the least type of thyroid malignancy which were found in a 55 years old female and in a 35 years old male respectively (Figure 1). Undifferentiated carcinoma had accounts significant proportion, 12.9%. Metastasis cancer to thyroid was also found in four patients, two of them were originated from the oropharyngeal area and the remaining spindle cell carcinomas were from unknown source.

Sex, duration of enlargement and enlargement size didn’t show statistically significant association with thyroid malignancy. However, age and type of enlargements have shown statistically significant association with thyroid malignancy. Older patients having an age of greater than 60 years and patients with solitary thyroid enlargement were more affected by malignancy compared to the reference age group, 11–20 years and diffused type of enlargement respectively (AOR: 10.96 (3.15-38.1; AOR: 8.82 (3.49-22.32) respectively) (Table 1).

**Table 1:** **Prevalence of tyroid malignancy at pathology laboratory of GUH from 2010–2013**

Variable		Non malignant N (%)	Malignant N (%)	COR (95% CI)	AOR (95% CI)
Sex	Male	171(92.4)	14(7.6)	1.05(0.56-1.94)	
	Female	613(92.7)	48(7.3)	1	
Age in years	0-10	22(95.7)	1(4.3)	1.5(0.18-12.76)	1.31(.156-11.78)
	11-20	231(97.1)	7(2.9)	1	
	21-30	236(91.8)	21(8.2)	2.94(1.23-7.04)	2.94(1.19-7.2)
	31-40	133(91.7)	12(8.3)	2.98(1.14-7.75)	2.68(0.99-7.22)
	41-50	91(91)	9(9)	3.26(1.18-9.03)	3.26(1.14-9.33)
	51-60	48(88.9)	6(11.1)	4.13(1.33-12.82)	4.97(1.51-16.33)
	>60	23(79.3)	6(20.7)	8.61(2.67-27.78)	10.96(3.15-38.1)
Size in cm	0-1.49	6(100)	0		
	1.5-1.99	3(100)	0		
	2-2.49	9(90.0)	1(10)	1.41(0.18-11.3)	02.87(0.22-15.98)
	3-3.5	6(85.7)	1(14.3)	2.11(0.25-17.83)	2.36(0.236-23.70)
	>3.5	760(92.7)	60(7.3)	1	1
Duration of enlargement in year	<0.6 years	58(93.5)	4(6.5)	2.48(0.27-23.1)	
	0.6-1 year	115(85.8)	19(14.2)	5.95(0.77-46.0)	
	2-5 years	365(93.1)	27(6.9)	2.66(0.35-2018)	
	6-10 years	160(94.7)	9 (5.3)	2.03(0.25-16.50)	
	11-15 years	36(97.3)	1(2.7)	1	
	>15 years	50(96.2)	2(3.8)	1.44(0.13-16.50)	
Type of enlargement	Solitary	78(78.8)	21(21.2)	9.154(3.75-22.35)	8.82(3.49-22.32)
	MNG	468(93.2)	34(6.8)	2.47(1.08-5.66)	2.52(1.07-5.93)
	Diffuse	238(97.1)	7(2.9)	1	1

COR: Crude Odds Ratio, AOR: Adjusted Odds Ratio.

## Discussion

In this study relatively lower prevalence of thyroid malignancy (TM) was observed, 7.3% (62/846). In contrast to this many report from United Kingdom (18.3%) [10], Romania (15.5%) [11], Ethiopia (11.5%) [12] and Pakistan (11%) [13] showed a little bit higher prevalence of TM. This may be due to the variations of the study design and sample size. Most of the above studies used only tissue biopsy sample which have greater sensitivity than FNAC thereb**y** it may increase the prevalence of thyroid carcinoma.

The prevalence of TM among MNG (34/502, 6.8%) was lower than a study conducted in India (10%) [14] and Italy (13.7%) [15] where as among solitary nodule, the prevalence of TM in the present study (21%) was higher than a study conducted in Pakistan (12.76%) and Sudan (13.5%). This may be due to the fact that 50% of solitary nodules found on palpation are actually part of multi-nodular goitre [16, 17].

This study depicted that PTC accounts the highest proportion of thyroid malignancies 28/62, (45.2%). In line with this finding, the leading type of thyroid malignancy that has been documented in different studies was PTC followed by follicular carcinoma [11, 18–20].

Follicular carcinoma was the second common type of thyroid malignancy in this study (29%). This is almost consistent with the finding obtained from Rawalpindi, Pakistan (25%) [14]. But different studies indicated that the proportions of FTC is in between 10-20% [18, 14]. This high proportion is probably due to high incidence of iodine deficiency goiter in the study area [21]. Hence iodine deficiency goiter is suggested to cause follicular carcinoma [8]. Hurthle cell carcinoma, which is a subtype of follicular carcinoma, also diagnosed in one (1.6%) patient having the age of 55 years. In line with this finding other study done in Romania also reported that HCC accounts 1.6% of the total type of TM [13] and the diseases is more likely occur in older patients [22].

Lymphoma was also found in 2/62, (3%) of the elderly female patients (>60 year) in this study. This finding is consistent with reports from other studies which reported primary lymphoma of the thyroid gland among old age (>50 years) females [23–25].

Due to the fact that data were taken in retrospective manner, the authors couldn’t determine the risk factors as well as associated clinical feature for thyroid malignancy like thyroid functions. Moreover, since data were collected from convenient FNAC technique (by excluding for neoplasm cases and inconclusive FNAC results), it may affect the result generated from this study. Tumors haven’t also staged according to its size, node metastasis and distance metastasis and this limits the information of this study. Future research with detail socio demographic information and clinical feature is crucial to determine the associated risk factors and clinical feature for thyroid malignancy.

## Conclusion

Prevalence of thyroid malignancy was lower than reports from other study areas but still considered significant and the leading type of malignancy was PTC followed by FTC. Thyroid malignancy has an association with type of enlargement and age of the patient.

## Abbreviations

FNAC: Fine needle aspirate cytology
FTC: Follicular thyroid carcinoma
GUH: Gondar University Hospital
HCC: Hurthle cell carcinoma
MNG: Multi nodular goiter
MTC: Medullary thyroid carcinoma
PTC: Papillary thyroid carcinoma
TM: Thyroid malignancies.
